# Unprofessional behaviours experienced by hospital staff: qualitative analysis of narrative comments in a longitudinal survey across seven hospitals in Australia

**DOI:** 10.1186/s12913-022-07763-3

**Published:** 2022-03-28

**Authors:** Antoinette Pavithra, Neroli Sunderland, Joanne Callen, Johanna Westbrook

**Affiliations:** Australian Institute of Health Innovation, Macquarie University, Sydney, Australia

**Keywords:** Unprofessional behaviour, Hospital, Employee satisfaction, Reporting, Whistleblowing, Speaking up, Organisational culture, Culture change, Patient safety, Staff well-being, Australia

## Abstract

**Background:**

Unprofessional behaviours of healthcare staff have negative impacts on organisational outcomes, patient safety and staff well-being. The objective of this study was to undertake a qualitative analysis of narrative responses from the Longitudinal Investigation of Negative Behaviours survey (LION), to develop a comprehensive understanding of hospital staff experiences of unprofessional behaviours and their impact on staff and patients. The LION survey identified staff experiences and perceptions related to unprofessional behaviours within hospitals.

**Methods:**

Two open-ended questions within the LION survey invited descriptions of unprofessional staff behaviours across seven hospitals in three Australian states between December 2017 and November 2018. Respondents were from medical, nursing, allied health, management, and support services roles in the hospitals. Data were qualitatively analysed using Directed Content Analysis (DCA).

**Results:**

From 5178 LION survey responses, 32% (*n* = 1636) of participants responded to the two open-ended questions exploring staff experiences of unprofessional behaviours across the hospital sites surveyed. Three primary themes and 11 secondary themes were identified spanning, i) individual unprofessional behaviours, ii) negative impacts of unprofessional behaviours on staff well-being, psychological safety, and employee experience, as well as on patient care, well-being, and safety, and iii) organisational factors associated with staff unprofessional behaviours.

**Conclusion:**

Unprofessional behaviours are experienced by hospital staff across all professional groups and functions. Staff conceptualise, perceive and experience unprofessional behaviours in diverse ways. These behaviours can be understood as enactments that either negatively impact other staff, patients or the organisational outcomes of team cohesion, work efficiency and efficacy. A perceived lack of organisational action based on existing reporting and employee feedback appears to erode employee confidence in hospital leaders and their ability to effectively address and mitigate unprofessional behaviours.

## Background

A growing body of literature has presented evidence demonstrating the negative impact that unprofessional behaviours amongst healthcare staff has on organisational outcomes, patient safety, and staff well-being [1–10]. Waterson et al. examined the enactment of patient safety culture across hospitals and highlighted the need to further explore the complex range of factors that impact patient safety culture within healthcare systems [11]. The relationship between human resource management practices, staff shortages, employee performance and patient satisfaction has also been explored by international studies, demonstrating the implications of organisational practices on employee and patient outcomes at hospitals [12]. Studies that have sought to demonstrate a link between staff well-being, patient safety and quality of care have called for further evidence to verify and elaborate how the metrics of staff well-being are associated with improved safety and quality [6, 13]. In studying the psychometric properties of teamwork and patient safety, researchers have also pointed out that future research needs to address how the “fault lines” in healthcare teams and leadership impact patient care and outcomes [14, 15].

Existing efforts to create comprehensive categorisation of unprofessional behaviour for medical professionals thus far, have largely focused on professional sub-groups such as medical students [16]. Recent scholarship has identified the need for better definition and conceptual clarity, and the need for categorisation of unprofessional behaviour in healthcare that is relevant to multiple professional groups [16–18]. Rogers and Ballantyne characterise professionalism within medical practice as spanning ethical and behavioural aspects in practical aspects of healthcare provision [19]. The current commonly accepted understanding of professional behaviour is characterised as being enshrined within institutional, local, and international codes of conducts that demand values-based behaviours anchored in respect, compassion, justice, integrity, and excellence [20]. Therefore, the converse of professional behaviour can be conceptualised as any manner of being, behaving or belonging within a healthcare organisation that negatively impacts other internal or external individual stakeholders, relationships, the cohesive environment at any level within the organisation, or organisational outcomes [18, 21]. While a growing body of work has identified the prevalence of unprofessional behaviours as experienced by groups of staff, such as nurses, physicians or medical students, further work is warranted to examine how the phenomenon of unprofessional behaviour unfolds amongst all staff groups across multiple contexts in healthcare organisations [2, 22–34]. Growing awareness of the negative impacts of unprofessional behaviours within the workplace, including within healthcare organisations, has led to developments in policy and regulation within Australia over the last two years to ensure psychosocial safety is considered a crucial factor within workplace health and safety management frameworks [35, 36]. As healthcare organisations implement these changes, there is a need for ongoing assessments of how staff experience unprofessional behaviour, its impacts and organisational factors that contribute to these experiences [37].

A recent survey was designed to understand the experience of unprofessional behaviours among hospital staff in Australia [38–41]. The survey invited participation from all staff at seven Australian hospitals enquired about the prevalence of 26 unprofessional behaviours, from rudeness to physical assault between staff [42]. In total 39% (*n* = 2009) of staff reported experiencing one or more of these behaviours in the previous week [42]. The survey also enquired about staff perceptions of the impact and organisational factors associated with both reporting and reducing these behaviours. In addition to these structured questions, the survey contained two opened-ended questions inviting staff to comment on their experiences of unprofessional behaviour in their hospital. The aim of this paper was to assess the descriptions of unprofessional behaviours provided by all staff groups within hospitals to understand the types of i) negative behaviours enacted by staff, ii) negative impacts on wellbeing and safety of human stakeholders, and iii) organisational factors that contribute to the prevalence of unprofessional behaviours.

## Methods

### Study design, scope and setting

The Longitudinal Investigation of Negative behaviour (LION) survey was administered to staff across seven hospitals in three Australian states between December 2017 and November 2018 to determine the baseline prevalence of unprofessional behaviours prior to an organisational intervention [42]. Two open-ended questions were included in the LION survey: 1) “Are there any specific instances of unprofessional staff behaviour that you would like to describe?”, and 2) “Are there any other comments you would like to make about staff behaviour in this hospital?”.

### Data synthesis and analysis

**Fig. 1:**
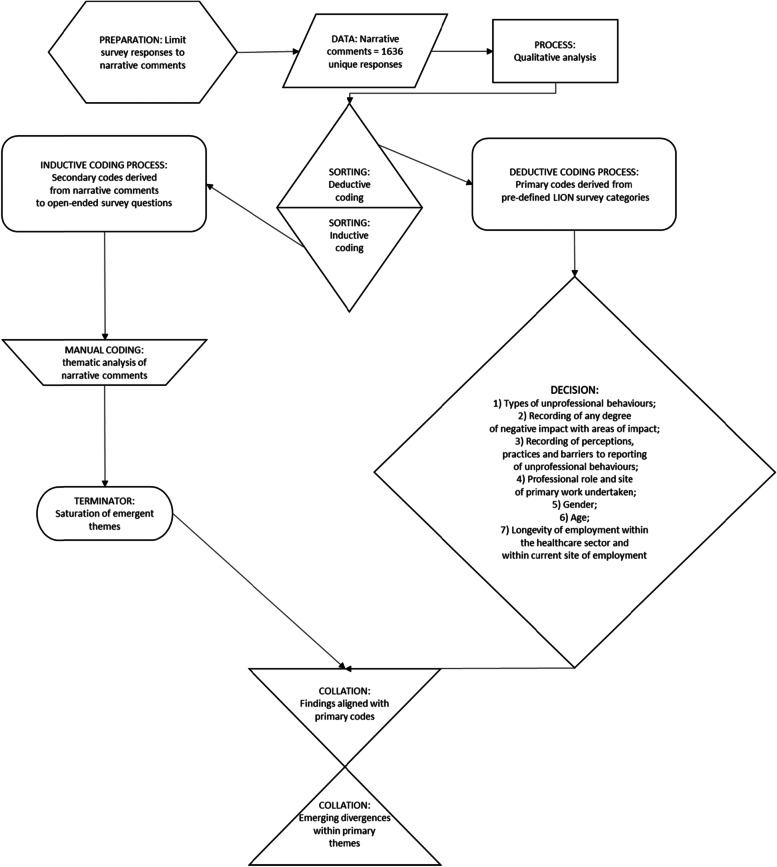
Directed Content Analysis performed on narrative responses to the LION survey

Narrative responses to the open-ended questions were imported into NVivo 12 (QSR International) to enable qualitative analysis using the Directed Content Analysis (DCA) method. DCA uses constructivist grounded theory to determine a preliminary coding scheme [43, 44]. The analysis was also informed by the themes presented within the closed-ended questions of the LION survey that offered definitions for types of unprofessional behaviours and perceived impacts on respondents [42]. The closed-ended questions in the LION survey spanned the following categories: (i) employee demographics, ii) types of negative behaviours experienced and frequency, (iii) degree and type of impact of the unprofessional behaviours experienced, (iv) self-identified speaking up skills, and (v) perceptions related to organisational factors. This part of the data analysis formed the deductive coding scaffold for the study of the narrative responses collected through the survey as outlined in Fig. 1. Inductive coding of narrative comments was performed by identifying descriptions about unprofessional behaviours provided by staff within narrative responses. Inductive coding was informed by the dimensions set out by the closed-ended survey sections, where questions covered the experience and perception of respondents in relation to 26 types of negative behaviours and impact types categorised based on staff, patient and organisational impacts [37, 38]. Deductive coding served to elaborate on the granular details pertaining to context and content of behaviours that have been reported as sub themes within the results. Systems-thinking-based approaches, particularly in relation to living systems and understanding work-related violence in hospital settings in Australia have informed the themes used to categorise the descriptions of behaviours provided by respondents [21]. Increased regulatory focus on the impacts of psychosocial risks on workplace wellbeing and safety have also informed our analysis and the need to highlight the linkages between themes – particularly the impact of organisational factors on employee psychosocial safety and wellbeing [45–47].

Two authors performed the qualitative analysis, where AP coded the entire set of narrative comments and NS independently coded over 20% of the narrative comments. Inter-rater agreement was over 90% and achieved through discussion till consensus was reached around the themes and sub-themes used to classify the comments. Coding was performed on all usable comments till themes were saturated, and all authors approved the final set of themes reported in the results. Quotes from narrative comments that have been provided within this article have been modified to de-identify professional groups, proprietary software names and individuals where necessary with asterisks (e.g., “***”).

## Results

### Respondent characteristics

From 5,178 LION survey responses, 32% (*n* = 1636) of respondents provided narrative comments to the two open-ended questions. Of these, 72% (*n* = 1183) worked in clinical roles, and 26% (*n* = 453) in non-clinical roles (Table 1). The profile of staff who provided comments to the opened-ended questions was very similar to the overall survey population (Table 1). For example, 11% of survey respondents were doctors, and 11% of comments were provided by doctors. Female respondents were slightly more likely than males to provide comments (e.g., 75% of surveys were completed by women, and 78% of the open-ended questions were provided by women). The high proportion of female respondents reflects the overall healthcare workforce which is 75% female [42].

**Table 1:** Characteristics of survey respondents who provided narrative comments in the two open-ended survey questions about unprofessional behaviour

Survey respondents who provided narrative comments (*n* = 1636)		Total survey respondents’ characteristics (*n* = 5178)
Role types*		
** Clinical roles**		
Medical	179 (10.9%)	546 (10.5%)
Nursing	735 (44.9%)	2248 (43.4%)
Allied Health	269 (16.4%)	795 (15.4%)
** Total**	1183 (72.3%)	3589 (69.3%)
** Non-Clinical roles**		
Management & Administrative	268 (16.4%)	822 (15.9%)
Support Services	157 (9.6%)	590 (11.4%)
Not specified	28 (1.7%)	177 (3.4%)
** Total**	453 (27.7)	1589 (30.7%)
** Age**		
18–24	91 (5.6%)	300 (5.8%)
25–34	429 (26.2%)	1567 (30.3%)
35–44	352 (21.5%)	1127 (21.8%)
45–54	374 (22.9%)	1097 (21.2%)
≥ 55	355 (21.7%)	983 (19.0%)
Not specified	35 (2.1%)	104 (2.0%)
** Gender**		
Female	1275 (77.9%)	3909 (75.5%)
Male	322 (19.7%)	1176 (22.7%)
Other / Not specified	39 (2.4%)	93 (1.8%)

^*^Role types:

***Clinical***

**Medical:** Medical Staff Specialist/VMO, Registrar, Resident, Surgical/Anaesthetic Staff Specialist/VMO, Career/Hospital Medical Officer/Medical Fellow, Intern

**Nursing:** Nurse Unit Manager or Associate NUM, Registered Nurse or Midwife, Graduate Nurse or Midwife, Enrolled Nurse, Clinical Nurse Consultant/Specialist/Educator

**Allied Health:** Allied Health, Clinical Services, Social, Welfare or Pastoral Care Worker

***Non-Clinical***

**Management & Administration**: Ward Clerk/Patient Services Clerk, Administrative Staff, Manager, Other Administrative or Managerial Roles

**Support Services:** Personal Care/Patient Services Assistant or Orderly, Cleaner/Environmental Services, Other Support Services Staff, Food Services, Engineering Services, Security or Tradesperson, Scientist, Laboratory or Research Staff

### Thematic findings about unprofessional behaviours

Participants provided a range of descriptions of unprofessional behaviours based on their lived experience and observed unprofessional behaviours within their workplaces in response to the two open-ended questions within the LION survey. These accounts were categorised according to three primary themes and the related sub-themes (Tables 2,3,4 and 5):

- Theme 1: Individual unprofessional behaviours.
- Theme 2: Negative impacts of unprofessional behaviours.
- 2a: Impact on staff well-being, safety, and employee experience.
- 2b: Impact on patient care and patient safety.
- Theme 3: Organisational factors associated with staff unprofessional behaviours.

### Theme 1: Types of individual unprofessional behaviours

The range of unprofessional behaviours described in Table 2 were behaviours that respondents stated had occurred between two individuals and were identified by verbal, non-verbal interactions, or behaviours demonstrative of a lack of positive values, such as unethical and discriminatory behaviours.

“Doctors yelling at nurses for no reason. Rudeness from doctors towards nurses such as sarcasm and belittling behaviours. Doctors showing sexually explicit photos / pictures on phones. Doctors being rude to nurses when called after hours with concerns about patients. Doctors calling nurses on their mobile phones whilst they are driving home after their shift and yelling at them bringing them to tears.”

**Table 2:** Theme 1: Types of individual unprofessional behaviours reported in the open-ended questions: “i) Are there any specific instances of unprofessional staff behaviour that you would like to describe? ii) Are there any other comments you would like to make about staff behaviour in this hospital?”

Type of unprofessional behaviours with examples provided by respondents in narrative comments		
Aggression	Non-Verbal Aggression	throwing things, hitting, slapping, breaking things, slamming doors, pushing, intimidating, stalking
	Verbal Aggression	yelling, shouting, screaming, swearing, raging, threatening
Incivility	Non-Verbal Incivility	sighing, ignoring, exclusion, ostracism, segregation, walking away, turning away, stonewalling, refusal to make eye contact, refusal to engage, withholding, being unresponsive, being hostile, being uncooperative, absence of socio-cultural niceties, lack of boundaries, refusal to acknowledge and practice appropriate boundaries, unwelcome pranks, invading personal space, unwelcome repetitive or casual physical contact, inappropriate gesturing, being unapproachable, unwilling to perform role adequately
	Verbal Incivility	interrupting, talking over, being dismissive, lying, misleading, vilification, rudeness, being overly critical, patronising, condescending, passive aggression, undermining, sarcasm, pedantic, belittling, humiliating, mocking, accusatory, finger-pointing, blaming, berating, telling off, scolding, being unreasonably demanding, gossiping, rumour-mongering, excessive complaining, bitching, inadequate socio-cultural niceties, back-stabbing, snapping, miscommunication, unclear communication, inconsistent communication, inappropriate humour
Unethical, discriminatory, or unjust behaviours	negative or exclusionary interpretation and enactment of organisational values that are at odds with patient or staff identity and dignity, abuse, misuse, inappropriate use of organisational resources, time, systems and process, sharing work-irrelevant social media posts, using personal apps such as dating apps during work hours, racism, homophobia, discrimination based on gender or sexual orientation, misogyny, sexism, anti-Semitism, Islamophobia, puritanical behaviour, over-protective unethical (shielding) tribalism, reactive tribalism (ethnic/ cultural cliques), bigotry, ageism	

In addition to overtly negative behaviours, comments also mentioned a wide range of behaviours that demonstrated lower grade negative behaviours encompassed within the concept of “incivility”, such as “working with headphones on”, hostility, not engaging in commonly expected socio-cultural niceties such as greeting colleagues, or other general forms of negative demeanours that were perceived as unprofessional.

“Just general incivility. People coming into work in a bad mood. Or people being passive aggressive about things not being done or not being done right…”

The normalisation of verbal and non-verbal incivility through facial expressions and body language within regular interactions was mentioned as a factor that contributed to creating a negative work environment.

"…Personal interactions with some team members and mangers can feel arduous as their reaction will be unnecessarily critical and feel like you are being constantly judged. Facial expressions and body language can be perceived that your performance will never be enough to satisfy...Witnessing other people get treated this way as well erodes the confidence of the team and affects team morale..."

Experiences of unprofessional behaviours such as eye-rolling and sarcasm within the context of performing work in high-risk work units were also described.

“I regularly experienced aggressive "over-ruling" and "eye-rolling" type behaviour when expressing clinical concerns about patient conditions in the ICU. It is very detrimental to confidence in reporting concerns when you are met with an offhand or sarcastic response. I feel it creates an environment where you are reluctant to report concerns for fear of being embarrassed.”

Extreme interpersonal negative behaviours that spanned harassment, bullying, assault, and discrimination appear to be well-understood and characterised as widely unacceptable. It is likely that well-developed policy, regulations, explicit directives across Human Resources (HR) and existing risk management systems contribute to this common understanding. Some of these types of behaviours described by respondents spanned themes related to sexism, sexual harassment and disrespect based on gender.

“I feel gender discrimination is part of everyday life at my institution. I am not offered the same level of respect or opportunities as my male counterparts. Comments about gender and pregnancy are a regular occurrence. Many of my female colleagues have experienced sexual harassment from prominent male colleagues and have never spoken out in fear for their careers.”

In addition to discriminatory behaviours based on sex and gender, a combination of other unprofessional behaviours such as unprofessional humour with discriminatory themes were also noted.

“… ‘Jokes’ in meetings about patients' ethnicity, social status, education level. – frequently…”

Descriptions of unprofessional behaviours extended beyond the experience of the primary behaviours themselves and went on to describe the flow-on effects when inadequate or inconsistent organisational action (or inaction) appeared to be perceived as unprofessional as well, resulting in compounding the impacts of individual interpersonal unprofessional behaviours, and making them a more pronounced phenomenon, at the organisational level.

“I hear a lot about the inappropriate behaviour of surgeons to patients and staff. One surgeon that we had as a patient, a few years ago now, was sexually verbally abusive to many staff members, including me, but nothing was done about it as the attitude was that he is a surgeon and therefore must be treated like a god. This hospital deals well with unprofessional behaviour if it is occurring from a general worker (they will be promptly taken to a disciplinary hearing), but it is very different if it is a manager or surgeon in which case that's ok, that's expected, no action required.”

### Theme 2: Negative impacts of unprofessional behaviours on staff and patients

The negative impacts of unprofessional behaviours described were clustered across staff (Theme 2a – Table 3) and patients (Theme 2b – Table 4). Direct impacts of experiencing unprofessional behaviours spanned negative outcomes related to psycho-social well-being, safety, and employee experience.

"I've seen people bullied and confidence squashed to the point they resign as an anxious mess. This seems like a popular strategy to move along the unwanted/unpopular… I've been told of reporting sexually inappropriate conduct, and because the perpetrator was a senior surgeon, nothing was done, and the complainant was disregarded…”

**Table 3:** Theme 2: Negative impacts of unprofessional behaviours. 2a: Impact on staff well-being and safety

Sub-Theme	Descriptions of types of negative impacts experienced individually by staff as a result of unprofessional behaviours
Impact on employee wellbeing and psychological safety	Emotional capacity negatively impacted resulting in dissatisfaction, anxiety, stress, depression or suicidal ideation
	Negative impacts on cognitive capacity of employees causing burnout, low morale, and disillusionment
Impact on employee experience related to relational and social workplace aspects	Experiencing a sense of isolation, lack of social cohesion and support at work
	Experiencing a lack of trust and confidence in colleagues and/ or leadership
	Experiencing diminished capacity to extend support and demonstrate respectful behaviour towards colleagues

**Table 4:** Theme 2: Negative Impacts of unprofessional behaviours. 2b: Impact on patient care and patient safety

Sub-Theme	Descriptions of negative impacts of unprofessional behaviours on how patient care is delivered
Poor manner and quality of care compromising patient experience and safety	ineffective care, ineffectual care, inefficient care, inadequate care, erroneous care, delays in care provision, timeliness of care impacted, lack of teamwork, lack of collaboration, lack of coordination, lack of person-centred care, ineffective and inadequate communication

The negative effects resulting from being exposed to unprofessional behaviours were described as being amplified by the unspoken codes of silence [48] surrounding widespread tolerance of negative behaviours, a sense of learned helplessness and disempowerment as a result of occupying a position that exposed victims to being abused by those in power, and conditional or inconsistent support [49]. Some respondents described the toll that being exposed to such behaviours has on their health and sense of well-being.

“My boss has left now - she used to hang up on me if I called in sick…Scream at me if I asked for a day off, refused to give me long service leave. I was scared of her; I wasn't the only one. Reporting would not have worked, being a boss!”

The effects, that staff who were exposed to these behaviours spoke of, included increased presenteeism (which refers to professionals being physically present and working, but under-functioning within their roles), ineffective teamwork, and an erosion of individual psycho-social and physical well-being over time.

"At this moment I am suffering from anxiety because of bullying: accusing…intimidating, abusing and ignoring… I have almost four months of sick leaves because I’m refused sick (leave)…As a victim I feel like no one cares… Some morning(s), I said to my (partner), ‘I don’t want to live…anymore, I want to disappear from all this negativity…for good. Enough is enough’, and my (partner) is very (concerned) because he is the only one (who) can see the (effect this has had) on me…”

Instances of negative behaviours, that had a direct or indirect impact on patient care (Theme 2b), ranged from staff treating patients and their families rudely and proving inadequate care to posting details about patients on social media. Examples of unprofessional behaviours in front of patients or in the process of providing patient care were provided.

“On one occasion in the last 12 months during a procedure with a patient, the staff member threw drapes onto the floor when they realised the solution used was something that the patient was allergic to. Rather than apologise and explain to the patient, wash the solution off and calmy recommence the procedure, the staff member picked the drapes off the patient and threw them across the room and on the floor. The patient was conscious and could hear and see this behaviour.”

Blatant violation of processes and procedures compromising patient safety and confidentiality were also described.

"I have seen staff uploading pictures in social media describing about the sick patient they looked after and including the pictures of the pump and patient they cared for.

The themes of these impacts on patient care and safety resulting from these behaviours are listed in Table 4. Comments indicated direct negative impacts on quality, manner, and safety of patient care, as well as indirect impacts as a result of poor teamwork and communication on the quality of patient care.

“… (unprofessional behaviours) this does have an impact on the quality of patient care employees are able to provide and can lead to poor staff behaviour, communication breakdown and further issues.”

Ultimately, cumulative episodes of unprofessional behaviours displayed by staff and their negative impacts over time appear to coalesce into a culture of blame and normalisation of negative behaviours within organisational cultures at hospitals.

“I was…yelled at by a doctor after being falsely accused of not doing my job- when the job was actually the responsibility of another staff member- which I politely explained to the doctor. The doctor then proceeded to make two other staff members cry as a result of verbally aggressive behaviour. This type of behaviour is unfortunately, not uncommon.”

Working within environments where normalised incivility was prevalent in addition to high degrees of professional stress was mentioned as a contributing factor to undermining staff well-being, performance, and safety.

“…It’s just making work a depressing place to be hence why there’s so many staff constantly taking sick leave because they’re over it.”

In describing the experience of a colleague who had resorted to taking stress leave resulting from a combination of negative workplace interactions and work-related stress, one respondent also added:

“…It is also noted that there have been a number of suicides of staff…it is very concerning if work stressors are a significant contributor."

“Repeat offenders” and senior staff were mentioned as perpetuating a culture that was disrespectful, and therefore diminishing efficiency of individuals, teams, and consequently impacting patient safety.

"I have witnessed a physician being highly unprofessional both over the phone and in person to several nurses on the floor I work. It has left the nurses shaken and I believe it will have an effect on patient safety if nurses don't wish to call the physician when needed.”

### Theme 3: Organisational factors associated with unprofessional behaviours

Organisational factors as a theme emerged repeatedly in response to descriptions of unprofessional behaviours, indicating that contextual institutional factors were perceived as inextricably linked to the positive or negative experience and perceptions of employees (Table 5). Inter-group unprofessional behaviours or instances were also included within this theme. Inter-group unprofessional behaviours are defined as behaviours enacted implicitly or explicitly by a group and not a single individual, which have a negative impact or are perceived negatively by staff. These behaviours were often associated with organisational factors that had contributed to unprofessional behaviours and were therefore categorised under this theme. The main sub-themes that emerged within this theme were:

i) Process, Performance and Practice
ii) Leadership and Management
iii) Learning and Development
iv) Remediation
v) Negative Clustering

**Table 5:** Theme 3: Organisational factors associated with staff unprofessional behaviours reported in the open-ended questions: “i) Are there any specific instances of unprofessional staff behaviour that you would like to describe? ii) Are there any other comments you would like to make about staff behaviour in this hospital?”

Sub-Theme	Types of negative organisational factors that contribute to the prevalence of unprofessional behaviours
Process, Performance, Practice	inadequate or under-developed operational direction, design and definition, ineffective or inefficient or inadequate quality management, workflow and workload, information and knowledge management practices, unsuitable and inadequate staffing, under-resourcing, impermanence of staff, inadequately trained staff, unfamiliar team members, high turnover, organisational or management and administration silos, top-heavy administration, conflicting drivers of growth, organisational priorities at odds with pre-requisites for staff well-being and patient safety, inaccessibility of channels for staff engagement, diffused responsibility, ineffective decision-making
Leadership and Management	negative leadership role-modelling of unprofessional behaviours, normalisation of unprofessional behaviours through tacit acceptance, lack of accountability, lack of transparency, hierarchical regard, conditional social status, micro-management, mismanagement, under-management
Learning and Development	disjointed, unrealistic or out-of-touch training, top-heavy learning models, unequal and inadequate staff development, absence of feedback loops
Remediation	inadequate organisational protection, institutional inaction, inadequate remediation, retaliatory action against whistle-blowers, inconsistent enforcement, inadequate disciplinary processes and standards, inadequate organisational monitoring
Negative Clustering	homophily, favouritism, cliques, tribe-mentality, negative sub-cultures, preferential treatment, inconsistent treatment

### Organisational factors related to process, performance, and practice

The complexity of multiple process or practice-based factors that influenced staff behaviour negatively were acknowledged and described by respondents. Lack of clarity in workflow processes and management appeared to contribute to work-related discontent.

“…Roles are blurred. Instructions are unclear and when you try to sort something out you are verbally attacked (or emailed) if something is not done 'correctly' even though you tried to seek out the 'correct' process…”

Conflicting priorities and diffused responsibility and the impact of these dynamics on the efficiency and quality of work was also noted.

“…Some enquiries to some departments/individuals go unanswered/ignored regularly. Not only does this impair my work efficiency, but also signals to me that I am not viewed as a customer/client of that department, or I and my work are seen as too far down…their priority list to bother with.”

A lack of accountability for the negative work-related dynamics appeared to further accentuate the normalisation of the interpersonal and group dynamics that were perceived as unprofessional.

“Power relations within each silo of disciplines…(e.g.) a clinician raising unprofessional behaviour of another clinician from a different discipline, reports up through line manager/stream manager, only for the reporting clinician to be made 'the problem'… shunt off to EAP (employee assistance services) …unprofessional behaviour continues… Line manager and line manager's manager continually demonstrate unprofessional behaviour. HR dept also demonstrates unprofessional behaviour, no one else to report to…”

Some other factors that contributed to the challenges that staff mentioned included issues related to risk management and personnel management practices.

“High staff turnover due to poor management by upper management. Particularly a concern when issues raised but no assurance that the issue(s) will be addressed.”

These challenges appeared to extend to issues such dealing with change, and the lack of sufficient resources for staff to perform work well, while being supportive of each other. This dynamic seemed to create low morale and possibly flow-on effects like individual unprofessional behaviours.

“I feel that there is a high level of stress among staff members at this institution related to increasing workload, need for change, poor implementation of new systems and poor communication. There seems to be less capacity for people to be supportive and low morale.”

### Organisational factors related to leadership and management

Respondents mentioned organisational factors impacting their ability to deliver appropriate care due to goals at the inter-personal, professional, service, and organisational levels being at odds. Comments noted the tension between what were perceived to be organisational goals such as remaining financially viable or profitable against the conditions they believed were required to provide person-centred care.

“As frontline workers, we value patient-centred care and the patient experience over the cost-cutting. Management are intent on viewing any staff who challenge such decisions as recalcitrant and therefore need discipline.”

Financial models and operational factors found multiple mentions within the comments.

“The work environment is toxic with male surgeons who bring in high revenue streams to the hospital (and) seem to be allowed to treat staff as they please despite the fact that staff have raised concerns, along with the fact that (some staff) seem willing to capitulate to these abusive ‘quirks’ of particular individuals for fear of losing their revenue streams.”

Profitability and financial viability of the hospital appeared to present a fault-line along which power is enacted, and solidified through the enforcement, or lack thereof, of protective policies and regulations.

“…Staff are not made accountable for their actions; policies are not enforced which promote best and safe practices. At times it appears that bad behaviour is rewarded rather than managed by the department managers.”

### Organisational factors related to learning and development

Teaching and mentoring junior staff was mentioned contextually in relation to multiple negative interactions.

“A senior nurse reported me to the NUM (nursing unit manager) for not knowing the entire instrument (set) instead of her going through each instrument so I can learn them while using it. (The) same staff nurse pick(ed) another nurse to do a count with her (theatre set-up) while I was already doing the count with her… Teaching must be done while doing the task and unnecessary reporting (of junior staff) without addressing or educating the person involved makes learning hard and the experience unpleasant.”

Comments that described interactions such as these drew attention to the efficiency of the clinical process and patient safety protocols that were being followed. However, uncivil behaviours that were enacted in the interest of preserving or improving efficiency appeared to in some instances to undermine the confidence and ability of junior staff to learn because of the manner of communication displayed.

“The culture here is poor, but largely because from my point of view there is excessive pressure placed on trainees to make up for shortfalls in hospital systems and processes…For example, Theatre late starts being ‘blamed’ on a registrar, or incorrect operative bookings being ‘blamed’ on a junior surgical trainee. Often these problems are out of control of the junior staff member, but they receive the burden of blame, and it leads to an intimidating culture.”

### Organisational factors related to remediation

Combined with job-based and organisational factors, unprofessional behaviours appear to be tolerated and internalised as normalised behaviour, creating a self-perpetuating cycle of negative behaviours, negative sub-cultures, and self-isolation among victims.

“I've had a staff member say she will only deal with me, not my colleague because of his inappropriateness toward her and his comments about her body. HR are aware of this, but she was not comfortable enough to make a formal complaint. She did not feel supported.”

The perceived incentivisation of unprofessional behaviours by the organisation, an unequal distribution of power, flow-on effects of internalised acceptance of unprofessional behaviours within certain contexts, and what appeared to be organisational inaction in stemming negative behaviours were considered causal to cementing unspoken norms and expectations related to unprofessional behaviours among staff.

"…comments from ***(professional group) about staff that are unfair and unprofessional…Constant passive aggressive comments that are uttered to demean and shame staff with management well aware of this behaviour and has been happening for years…Despite staff bringing concerns to management staff seldom see a positive outcome and are rarely informed if their concerns are being addressed. With staff being reduced to tears for fear of working (with)*** surgeon… The environment is toxic.”

The “seriousness” of behaviours appeared to be a factor in determining whether staff would report these incidents using formal mechanisms. This appeared to be the case particularly when hierarchical dynamics had also informed these interactions.

“If very senior staff are perpetrators of problematic behaviour and they are a manager or a person the junior staff member reports to, it appears very difficult for reporting to be initiated unless it has become very serious.”

### Negative clustering behaviours within specific work environments and roles

The design of organisational structures and distribution of power invested within certain professional roles also appeared to trigger aggressive or abusive behaviours from certain staff within some environments repeatedly. Behaviours on the lower spectrum of severity such as incivility appeared to intersect with negative intergroup behaviours as well, thus contributing to negative sub-cultures over time because these behaviours seem to be considered more commonplace, diffuse, and thus, more difficult to address. Operating theatres were mentioned as environments that foster a climate that lent itself to higher degrees of unprofessional behaviours, as well as more extreme forms of unprofessional behaviours.

“I often find the operating theatres an unpleasant place to work due to the behaviour of several staff members that seems to be fostered by within an insular unit such as operating theatres.”

Professional stressors related to surgical processes and procedures appear to activate or elicit authoritarian and hierarchical modes of interacting between inter-professional groups. This may be associated with staff lapsing into verbal or physical unprofessional behaviours.

“Offensive language and threatening language (used)… ‘I'm reporting this to your boss’, ‘Do you know who I am?’ used by surgeons when they do not get their way or there are difficulties with examination requests or in theatre cases... Nursing staff can be very rude and aggressive to radiographers when asking for crucial patient safety questions to be completed or filled out properly.”

Such instances may be associated with self-perpetuating cycles of negative climate within specific environments and entrenched practices that may come to be seen as reflective of professional sub-groups.

“Some (surgical medical staff) in theatres are still very rude to nurses - every week I would witness a surgeon raising his voice, yelling, throwing things out of anger and impatience…”

Further, within these professional sub-groups, certain sub-specialties were named by multiple commenters, noting that professionals who belonged to these clusters appeared to demonstrate a greater pre-disposition to displaying unprofessional behaviours.

"Aggressive swearing from (a) surgeon in theatre if (there was a) perception of difficulty, raised theatre temperature, or) having to wait for anything…Belittling negative and passive aggressive commentary from surgeon (was directed) to (the) entire team of specialty. (The) staff (were) so demoralised they were unable to work in (the) theatre. Some staff reduced (their) hours and changed workdays to avoid this individual. Others refused to work with them.”

Spatial configurations that involve multiple staff at varying levels of experience and expertise may introduce conflicting roles and expectations that increase the complexity of the primary task and patient care.

“(A) ***(sub-speciality) surgeon (was) yelling at theatre tech for being unable to find (the) correct piece of equipment. Another *** surgeon (was) yelling at scrub nurses and throwing instruments because certain equipment (was) unavailable – (the) surgeon did not check availability before set up. A third *** surgeon (was) putting pressure on theatre staff to rush starting a case because he had another list in another hospital to start…In my experience the worst behaved staff at *** are the surgeons…”

The associating between negative clusters and patterns based on clinical environments such as operating theatres or working within surgical specialties by extensions were seen as contributing to a psychological and physically unsafe working environment.

“…I felt unsafe in that environment… In the theatre environment at this hospital, I have been sexually harassed verbally and bullied by senior staff…”

Ultimately, descriptions of negative behaviours noted the intrinsic link between unprofessional behaviours and the challenging circumstances inherent to working in healthcare. Positive acknowledgments of exemplary behaviour were mentioned within comments specific to certain hospital sites and work groups. These comments were notable as respondents commented that despite exposure to negative behaviours, ultimately, positive experiences did contribute to employee satisfaction and engagement at work.

“I have been blessed to work on a ward with an amazing and supportive...staff. I am happy the minute I walk through the doors no matter what’s going on in my life. I love my work and the people who work there in all the different jobs about the place.”

## Discussion

Our results offer insights into a variety of views from a broad range of hospital staff across varying roles, departments, and diverse employment arrangements. Using an approach based on systems theory is appropriate to understanding the narrative comments because this approach has allowed us to preserve and report on the complexity of interactions between elements that result in individuals’ experience of organisational culture and unprofessional behaviour, rather than reducing these lived experiences to statistical artefacts that can be neatly delineated. The need for localisation and customisation of training and remediation mechanisms may be warranted owing to the heterogeneity in how unprofessional behaviours are experienced across professional roles and specialities as well as by those with different demographic characteristics [50]. The narrative comments analysed within this study offer a rich insight into the complex relationships between individual staff behaviours, group structures, structural inadequacies, and implicit expectations that contribute to the phenomenon of unprofessional behaviours among hospital staff. These findings further elucidate results presented by previous researchers related to the complex inter-relationships between multiple inter-personal, individual, and organisational factors in giving rise to the phenomenon of unprofessional behaviour [15, 23, 26, 51–59]. Organisational and sector-related socio-cultural and contextual factors (such as negative, internalised sub-cultures and pervasive incivility) also seem to have a significant influence on the experience and under-reporting of unprofessional behaviours that has been identified by previous studies [5, 6, 60–62].

While confirming the findings of previous studies that have identified the complex interactions between organisational and individual staff factors in the emergence of unprofessional behaviours among hospital staff, our study also provides further clarity around these themes. The identification and categorisation of specific behaviours displayed by individual hospital staff members, the prevalence and proliferation of these behaviours within specific spatial environments, as well as their relationships to organisational structure, leadership and management factors are a significant addition to the literature related to healthcare organisational behaviour and culture. Consequently, the range of perceived negative impacts of these behaviours on staff satisfaction, well-being and safety, and not just patient safety and well-being has not previously been reported across a similar range and scale of multiple staff roles, groups, and hospital services, to the best of our knowledge. Our study presents a synthesis of clinical as well as non-clinical staff from across a large-scale multi-site cohort, demonstrating the spread of unprofessional behaviour among hospital staff as a pervasive problem with several common features. This presents a promising avenue for ongoing culture change interventions to focus efforts.

Existing literature does not appear to have sufficiently elaborated the prevalence of divergent experiences of unprofessional behaviours, therefore, making the process of designing suitable interventions challenging. From our findings, training staff to collectively understand what unprofessional behaviours mean and establishing consistency in expectations and interventions across professional groups and hospital environments might be a foundational step towards achieving improvements in staff perceptions of positive organisational culture. In addition, hospital policies and governing mechanisms as interpreted by employees appear to lack articulation around the challenges that can give rise to, as well as result from, unprofessional staff behaviours. An erosion of employee confidence in hospital leaders and their ability to effectively address and mitigate unprofessional behaviours, associated with the organisational factors reported by respondents, could undermine hospital management efforts to improve staff working conditions and eventually, efforts to improve organisational culture. Solutions such as implementing responsive feedback loops between staff and management, and co-constructed and cross-sectional modules for all hospital staff to understand acceptable and inappropriate behaviours may present practical approaches for hospital management to gain employee confidence and remedy the widespread problem of unprofessionalism among their staff.

### Limitations

Pre-defined categories within the closed-ended questions of our survey may have limited the emphasis of responses by pre-determining the categories of behaviours that we sought to understand from responses to the open-ended questions. Further, the broad phrasing of the open-ended questions resulted in a wide range of comments that reflected multiple aspects of organisational culture, unprofessional behaviours as well as impacts, and the interactions of all these elements with each other. A gap in our study design may have resulted from the decision to perform qualitative analysis on the comments after primary quantitative analysis had been conducted, rather than including more specific open-ended questions that corresponded against each of the categories we have investigated and reported on. In addition, the use of directed content analysis methods may have resulted in positive bias in coding themes within the data. Finally, the qualitative findings reported within this article are based only on textual responses to the open-ended questions within the LION survey. Therefore, further elaboration of our findings is recommended through interviews and focus groups. These additional research methods are recommended across multiple sites and professional groups to test the validity of our insights against other contexts.

## Conclusion

Unprofessionalism in hospital settings is diverse in terms of how it is perceived, understood, and experienced by hospital staff, across professional and personal demographic categories. Perceived lack of organisational action to contain and address unprofessional behaviours appears to have a significant effect on the internalisation of unprofessional behaviours as professional norms, resulting in underreporting and ineffective remediation of these behaviours.

Complex factors that include internal sub-cultures because of the manner of power distribution across the organisation may play a major role in how prevalent unprofessional behaviours are among certain staff groups and within certain environments. The challenge of addressing these factors is complex, but not insurmountable. The phenomenon of unprofessional behaviours among medical professionals requires further study to identify effective tools that do not merely address behavioural challenges among individuals, but also address the systemic, structural, and organisational gaps that have led to these behaviours. Some such gaps are perceived organisational inaction, inconsistent enforcement of remediation, and unequal distribution of power, and the lack of integration of expectations for respectful behaviours at the level of human resource management and career progression. Concerted efforts not just by researchers, but more importantly, by professional bodies, and hospital administration and management, are required to ensure that flow-on negative effects on patients and staff can be stemmed sustainably. The benefits from such improvements would have far-ranging positive effects across all stakeholder groups including staff, patients and wider healthcare organisations and networks.

## Data Availability

The data generated and analysed during this study are not publicly available to protect the privacy of survey respondents. All relevant de-identified supporting material reported within this study has been provided within the manuscript.

## Abbreviations

LION: Longitudinal Investigation Of Negative behaviour survey
DCA: Directed content analysis
